# Obstacles to the development of the organic food market in Poland and the possible directions of growth

**DOI:** 10.1002/fsn3.704

**Published:** 2018-06-19

**Authors:** Magdalena Jarczok‐Guzy

**Affiliations:** ^1^ University of Economics in Katowice Katowice Poland

**Keywords:** consumer, organic food, the European Union, the Polish organic food market

## Abstract

The general purpose of the work was to assess the development of the organic food market in Poland and to indicate the reasons for the limited development of this market from the marketing point of view. To carry out the research, a survey has been conducted on a sample of consumers to check the attitude of Polish consumers to organic food and to the marketing elements associated with this food. Consumers’ knowledge about this food in Poland and shopping habits have also been examined. The reasons for the limited development of this market in Poland have also been diagnosed. The survey conducted on a sample of consumers shows that almost all the people (94%) have heard about organic food but the reason for the lack of interest in organic food by the consumer is ignorance about its labeling (20%) and high prices (48%). The research has been made on 1159 customers from Silesian Voivodeship. In selection, the snowball method has been used as a selection method, then the statistical analyze has been made with using Excel.

## 1 INTRODUCTION

As a part of sustainable development in the agriculture sector, there is integrated farming which is a method between conventional and organic farming methods. It reinforces the positive influences of agricultural production and reduces negative impacts. Integrated farming makes a vital contribution to sustainable development by adding consideration of economic, ecological, and social objectives to the essential business of agricultural food production (Bosona & Gebresenbet, 2018). In Poland, the organic food market is developing very dynamically. Poland is also perceived as a country of the European Union with a huge potential of organic farming and organic food market. Despite the numerous state support programs, Poland still maintains its midlevel position in the rankings of organic farming areas and the number of organic farms. Therefore, it is important to answer the following question: what inhibits the development of the Polish market and organic farming and what measures should be taken to improve the situation of this food industry? According to the latest research in Poland, there are more and more conscious consumers buying organic food from the conviction about its properties. The marketing research is one of the ways to recognize the market situation. The market structure and effectiveness of marketing tools commonly used in this industry should be analyzed. Understanding the exact characteristics of the organic food consumers and their opinions will be useful in describing the target segment interested in purchasing bio‐food and in planning marketing activities such as appropriate forms of promotion. At the outset, it should be noted that organic food as a “product” in the context of the marketing mix will not be fully considered in this work. Such food is not often packaged in neatly designed packaging, but sold in bulk. In the case of such products as meat, bread, fruit, or vegetables, the packaging has only preservative functions to protect food against damage during transport. However, in the case of dry products, we deal with a packaging that should draw the customer's attention primarily by a clear label with the logo of the organic origin of the product and with the list of ingredients. Therefore, this issue has been omitted in this work. The other elements of the marketing mix have been analyzed: promotion, price, and distribution. Characterizing of this unexplored area has become the reason for writing this article.

The general purpose of the work is to assess the development of the organic food market in Poland and to indicate the reasons for the limited development of this market from the marketing point of view.

The work involves the methods of statistical analysis, synthesis, and induction.

## 2 LITERATURE REVIEW

### 2.1 The organic food market and the essence of organic agriculture

#### 2.1.1 The concept of organic food

Organic food means products which in accordance with the EU law have a certificate described in the right way on the packaging by the EU logo and the address of the certification body. Organic food is confused with what on the market has been functioning for years as “healthy food”—natural food which has nothing in common with the ecological process of production. The term “żywność ekologiczna” has been legalized as the equivalent of the English “organic food,” while in the European Union there are three synonyms: organic agriculture = biological = ecological. To be fully accurate, it would be better to talk about food from the organic farms, while the term “organic food” has already started to function as a proper name (Wieczorkiewicz, 2008).

Ecological food is one that is produced without the use of chemical pesticides and fertilizers and which does not contains synthetic hormones. Produced according to legally defined methods, and its composition must to comply with general laws and regulations quality of food (Kuran & Mihić, 2014). Organic food fulfills the requirements of consumers on quality food, where mineral fertilizers, hormones, GMO's, and pesticides are not used during its production, the best before date is not prolonged artificially during its secondary processing and the taste, color, and aroma are not chemically improved (Živělovă & Crhovă, 2013). There are no used antibiotics. The term antibiotics encompass a wide range of chemical substances that are produced naturally, semisynthetically, and synthetically and are used to inhabit bacterial growth or kill them. They are categorized based on their effects as either bacteriostatic or bactericidal, and on their series of efficacy, as narrow or broad‐spectrum antibiotics (Manyi‐Loh, Maphweli, Meyer, & Okoh, 2018).

The foods of animal origin are highly perishable due to high nutritional content, moisture, and neutral pH. These foods require proper preservation to maintain quality and safety. Failing which leads to human illnesses and disease outbreaks. These food borne illnesses are serious and costly public health concern worldwide. So to maintain the quality and safety of foods various measures are generally adopted in food industry that is good manufacturing practices, good hygienic practices technic. But preservation of food by a suitable means is the key of food quality and safety (Singh, 2018).

Proponents of organic agriculture frequently point out the many drawbacks of conventional, industrial, agricultural practices. They claim a range of benefits which organic agriculture purportedly provide (Tal, 2018):

- organic agriculture eliminates chronic and acute exposures to toxic pesticides among farm workers, consumers as well as surrounding aquatic and terrestrial ecosystems;
- organic produce has higher nutritional value with greater vitamin and mineral content, it is also argued that organic produce tastes better due to its higher sugar content and keeps longer due to its high metabolic integrity and superior cellular structure;
- organic farming fosters healthy soil and soil microbiota facilitating the availability of nutrients to plants;
- organic agriculture avoids genetic mutations and development of immunity among insects, reducing the pest outbreaks that pesticide us can unintentionally foster;
- by eliminating the expense of many inputs‐including insecticides, herbicides, and synthetic fertilizer—organic agriculture cost less and is economically competitive;
- by relying on inputs that exist in nature, organic agriculture offers a more harmonious orientation toward the natural world and, as such, constitutes a preferable ethical strategy for humankind.

**Figure 1:**
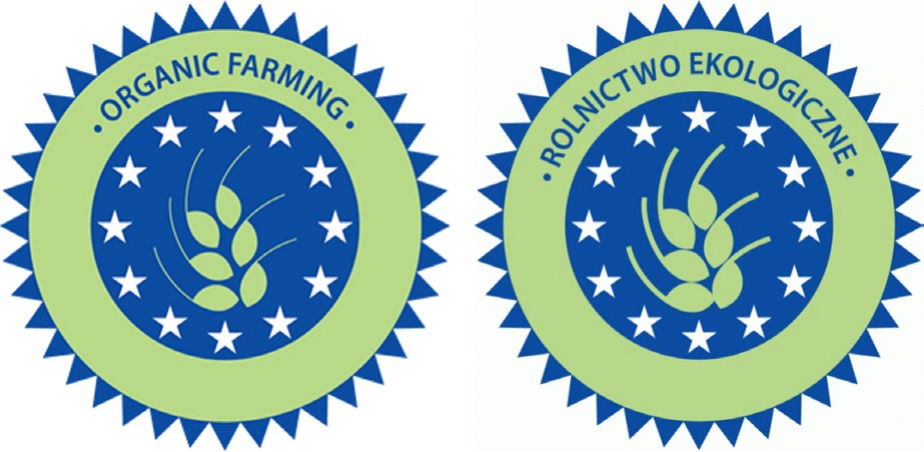
The Logo of organic food in accordance with the EU regulations valid until June 30, 2010. *Source:* http://ec.europa.eu/agriculture/organic/eu‐policy/logo_pl download date 23/02/2018

**Figure 2:**
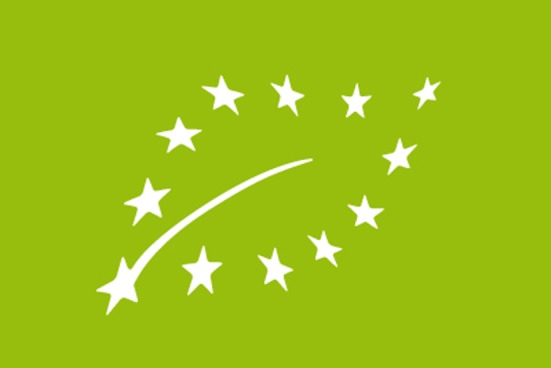
The organic food logo consistent with the EU regulations valid from July 1, 2010. *Source:* http://www.minrol.gov.pl/Jakosc‐zywnosci/Rolnictwo‐ekologiczne/Logo‐rolnictwa‐ekologicznego download date 23/02/2018

The European logo identifying organic food valid until the June 30, 2010, is shown in Figure 1 next to the Polish version of the European label for organic farming products. The new logo valid from the July 1, 2010, visible on all the packaged organic products made in the EU countries that fulfill the strict standards of organic farming is shown in Figure 2.

#### 2.1.2 The organic food market

There were large differences between Member States in terms of level of development and production systems have the advantage of complementarily agriculture, especially from France, which have surplus grain and Germany, poor food, Italy Mediterranean products have comparative advantages (Gheorghiu, Gheorghiu, & Jacob, 2014). In 2007, organic food production in Poland was only 1–1.5% of the volume of commodity production. According to the Inspection of Trade Quality of Agricultural and Food Products, the number of organic producers on December 31, 2015, decreased to 23,015 in comparison with 2014, when there were 25,277. This is a decrease of 9.49% which also translates into a decline in the number of organic producers operating in the field of agricultural production, a decrease in 2015 by 10.28% compared to 2014. However, there is an increase in the number of processors of organic products and fodder and/or yeast production from 484 in 2014 to 562 in 2015. Also, there was an increase in the number of organic producers active in the supply of certified seed and vegetative propagating material by as much as 47.30 %—from 74 in 2014 to 109 in 2015. When we look at the change in the total area of ecological arable land in 2015, compared to the previous year, we see a decrease of 11.73%, which gives us the current number of 580 730.03 ha (2015). It is important to notice the greater decline in the area of ecological arable lands in the conversion period, a decrease of 22.74% in 2015 compared to 2014. As for the decline in an ecological arable land after the conversion period, it is 9.71% (from 555 898./36 ha in 2014 to 501 924.90 ha in 2015) ( http://ekoarka.com.pl/kolejny-spadek-liczby-eko-gospodarstw-i-powierzchni-eko-uzytkow-rolnych/ entry date of download 24.02.2018).

## 3 METHODS

The survey on consumers has been conducted with the help of the Internet using the ebadania.pl panel and simultaneously in the paper version distributing the survey among the inhabitants of the Silesian Voivodeship. The research sample has consisted of adult consumers of grocery stores from the Silesian Voivodeship in the number of 1159 people.

The subject scope of the study includes:

- knowledge of the labeling of organic food and the characteristics of its manufacturing process that differentiate it from conventional food
- circumstances accompanying the purchases of organic food and factors hindering the purchase
- real motives for buying organic food
- demand for organic food among Poles
- the perception of prices, promotion, and distribution on the organic food market by consumers.

The objectives of the research, which are to serve the main purpose of the article, are as follows:

- assessment of Polish consumers’ behavior in relation to organic food
- identification of the reasons for the purchase of organic food
- determining the state of consumers’ knowledge about the characteristics and labeling of organic food.

## 4 RESEARCH AND DISCUSSION

### 4.1 Consumer profile

**Figure 3:**
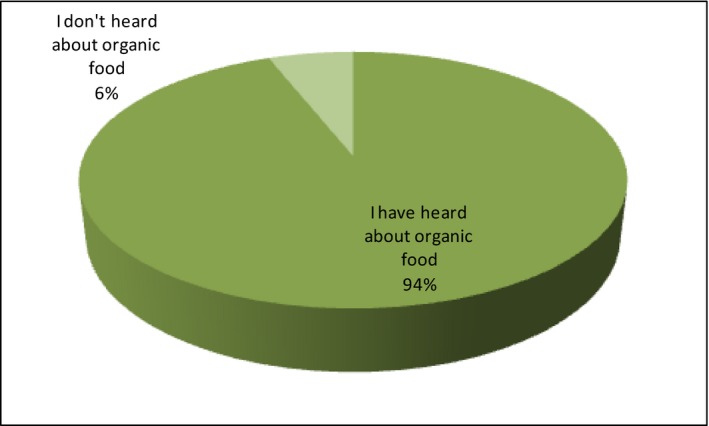
Knowledge of term “organic food”

At the beginning of the survey, the respondents have been asked about the general attitude toward organic food. In the first question, they are to declare “have they ever heard of organic food?” The vast majority of respondents agree that such a term is known to them (94%). Only 6% of respondents have never come across this concept. Figure 3. illustrates this data.

To explore deeper the analysis of the results, the answers have been compiled according to such parameters as age, income, education, and the respondents’ place of residence. The detailed analysis shows that consumers who have ever heard about organic food are people aged 36–45 (24.4%), with incomes 1501‐2500 PLN (30.9%), with secondary education (42.2%) and city residents up to 100,000 inhabitants (35.2%). A lot of rural residents have reported knowledge of the concept of organic food (34.2%). Among people who have not heard about bio‐food are young people at the age of 18–25 (2.2%). The analysis of 6% of negative responses has been omitted due to the small number of indications, because a deeper analysis of the structure of the research sample of this variant could lead to erroneous conclusions.

**Figure 4:**
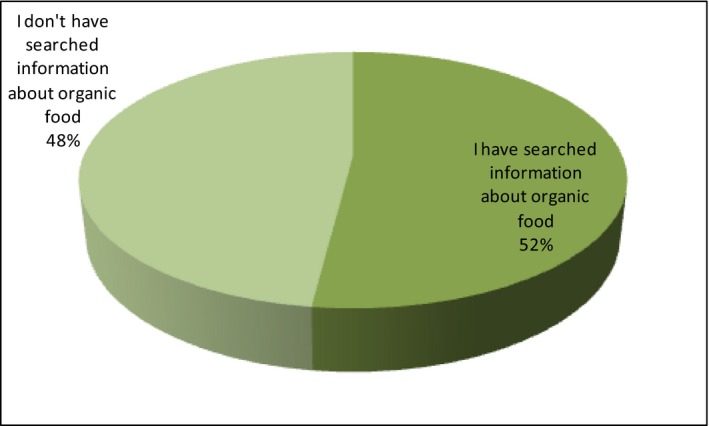
Searching information about term “organic food”

Then, the respondents have answered the question if they have ever looked for information about organic food. Here, the answers are almost equal (Figure 4).

The majority (52%) answer that they are interested in searching the news about bio‐food, but as many as 48% of respondents admit that they have never done it. Among the respondents seeking information about organic food, the largest group are city residents up to 100,000 inhabitants (19.9%), with secondary education (23.5%). The residents of the village are divided into two teams—18.5% who are not looking for information about organic food and 18.8% who are exploring their knowledge. The majority of respondents with vocational education admit that they do not look for information about bio (8%), only 3.7% of this group is expanding their information resources.

**Figure 5:**
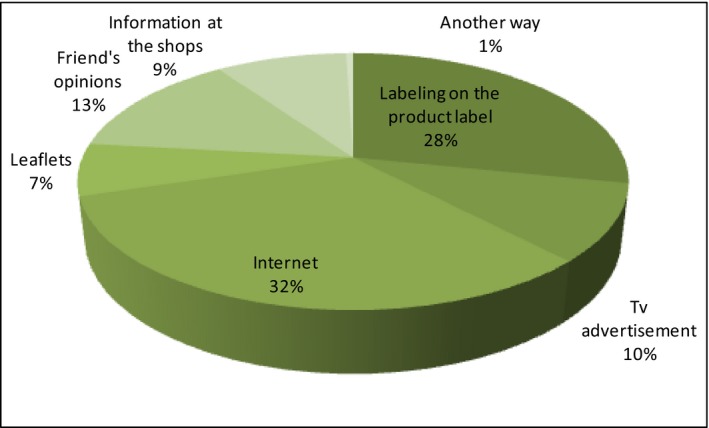
Sources of information about organic food

Next, the respondents have been asked to provide a source where they would look for information about eco‐food (Figure 5).

In the first place, consumers put the Internet (32%) and the label on the product (28%). These two dominating places of searching for product information are undoubtedly indicative of where it is the best to put logos and other information to reach a potential consumer. Therefore, The Internet and the label can be treated as the basic forms of a product promotion. A lower recognition is received by friends’ opinions (13%), TV advertising (10%), information in the store (9%), and leaflets (7%). As another way, the respondents mention: healthy food fairs, scientific research, TV programs, knowledge of specialists. Most people who have ever looked for information about bio‐food use the Internet (18.4%), label (16%), and friends’ opinions (6.9%). The second part of the respondents, who have not received information about organic food yet, also indicates the Internet (14%), and the label (12.1%) as the best source of data.

**Figure 6:**
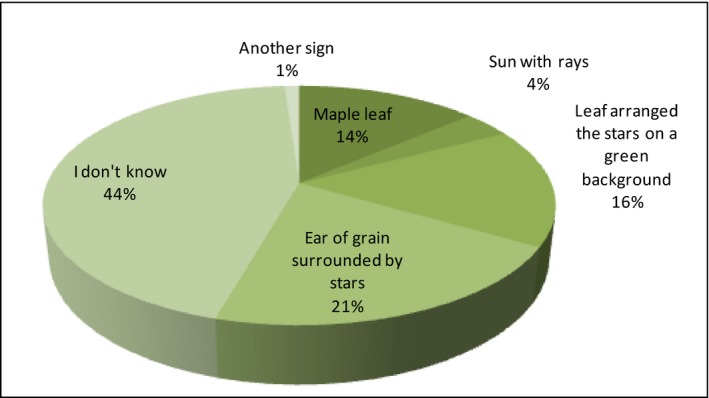
Knowledge of an eco‐label logotype

Knowledge of an eco‐label logotype labeling has been examined by asking the respondents the question in which they have had several options to choose (Figure 6):

In the first section of this work, the correct logo applied to organic food has appeared. Until the end of June 2010, it was an ear of grain (Figure 1), since the July 1, 2010, a new logo—a leaf made of stars on the green background (Figure 2) has been applied. It is particularly alarming that as many as 44% of respondents admit to an ignorance of this sign, and 19% recognize the logotype incorrectly. The correct answer is a leaf composed of stars on a green background, which has been chosen by only 16% of the respondents. It can be concluded that this group deliberately recognizes organic food on store shelves. The study deliberately places a version of ear of grain surrounded by stars, because it is a logo valid until 2010 and could still be remained in the consciousness of numerous consumers. The study shows that the ear of grain surrounded by stars has been considered as an eco‐food logo by 21% of the respondents. The respondents who correctly recognize the sign are people with secondary education (8.1%) aged 35–46 (5.3%). In an interesting manner, the ear of grain has been chosen by the youngest respondents (6%). It is clear that, young people easily associate graphics with organic food. People who knowingly admit to ignorance are the respondents both with secondary education (18.7%) and high education (15.7%) and those in the 26–35 age group (11.7%).

The detailed knowledge of respondents about products of organic origin has been analyzed in this part of the study. First, the respondents have been asked about the properties of the manufacturing process and then, about the characteristics of food itself. Consumers have had to choose from all the available variants of several correct sets. In the first question, respondents have been given the following options for selecting the answers characterizing the process of producing eco‐food:

A It contains at least 95% ingredients produced organically
B It is produced and stored without preservatives
C It may contain artificial colors
D It may contain genetically modified organisms (GMOs)
E It comes exclusively from certified organic farms
F All of the above answers are correct
G I don't know

**Figure 7:**
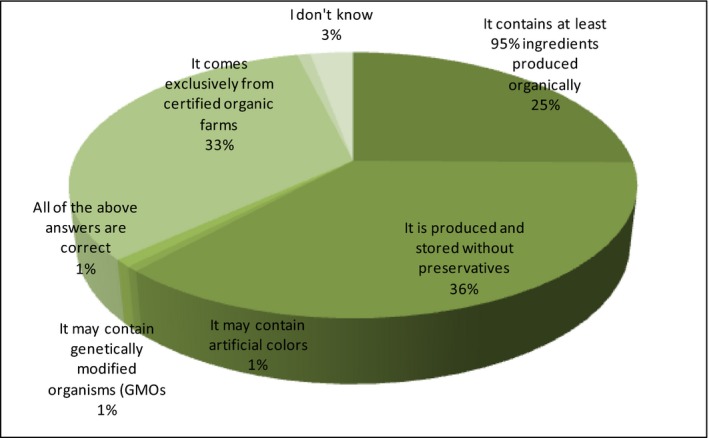
Knowledge about the organic food producing process

The correct answers are A, B, and E. The appropriate variants are successively: 25%, 36%, and 33% of the selected answers. The vast majority of respondents correctly identify the properties related to the production of organic food (Figure 7).

In turn, the respondents have been asked about the characteristics of a produced organic food. The possibilities to choose for the respondents were as follows:

A Higher content of vitamins
B Lower content of impurities
C Lower nutritional value
D No preservatives
E All of the above answers are correct
F I don't know

**Figure 8:**
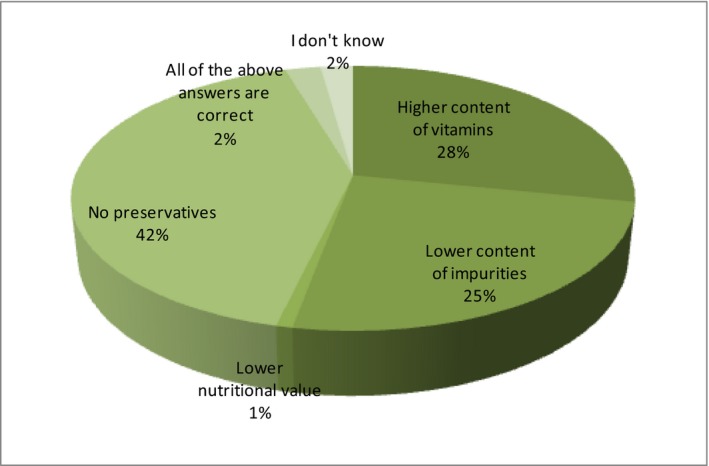
Knowledge about the properties of organic food

The correct variants are A, B, and D. As in the previous question, the respondents show knowledge about the properties of organic food (Figure 8).

That is evidenced by the correct answers of 28% for a higher content of vitamins, 25% for a lower content of impurities and 42% for lack of preservatives. Such awareness of the respondents undoubtedly may indicate their trust in bio‐food. However, in order to check a consumer confidence expressed in the purchase of eco‐food, one should ask the same respondents.

### 4.2 Consumer buying behavior

In this subsection, the declared behaviors of entities have been examined. People in the research sample have been asked if they have bought organic food during the last year. Just over half declare that they have purchased organic food (54%).

In order to get to know the exact characteristics of the segment of people buying and not buying organic food, an analysis has been carried out in terms of education, age and net revenues of the respondents. Consumers of eco‐food are people with a secondary (25.6%) and high education (21.6%), aged 26–45 years (29.9%), with revenues 1501–2500 PLN (18.4%). Whereas the group of respondents who do not consume bio‐food is also characterized by secondary (19.2%) and high education (16%). They are young people at the age of 18–25 (15.2%) and with reasonable revenues (14.1%). In the group of people with the lowest revenues, there are definitely fewer consumers of organic food (6.2%), similarly is in the next range of PLN 1501–2500 (10.8%). It is worth noting that people who have never bought bio‐food dominate so clearly only in the youngest age group. Perhaps it results from the fact that they lack experience in shopping or are hostile to this type of goods. The profile of organic food consumer has also been analyzed in terms of knowledge of the organic logo, perception of the price level and the preferred form of promotion for organic products. Of those who have ever bought organic food, definitely the largest number of respondents admit that they do not know the organic logo (18%), only 12% recognize the sign correctly, and 14% indicate the nonobligatory labeling of organic food. In the group of people who are not consumers of organic food, 26% are people who do not recognize the organic logo, and only 4% manage to correctly assign a sign to organic food. Among the consumers of organic food, in most cases there is a belief that the price level in the market is high (23%) and average (21%). Whereas people who do not buy organic food also think that prices are high (19%) and 16% have not expressed their opinions. Very few respondents consider organic food prices as too low. The consumers of organic food agree that a reduced price (31.3%) is the form of promotion encouraging to buy this type of products. In an interesting manner, the same opinion is expressed by a large percentage of people who do not buy bio‐food (24.6%). A special attention should focus on the promotion “2 for the price of 1” for both consumers of organic food (8.4%) and consumers not buying this food (7.4%).

**Figure 9:**
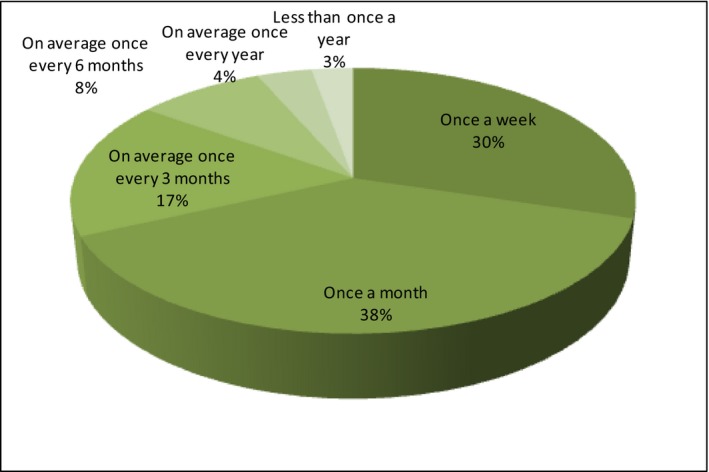
Frequency of purchases

Further questions regarding the purchase of organic products have been asked to a group of respondents who have ever bought organic food. At first, the frequency of purchases has been examined then, the type of purchased food and the preferred place of purchase. Regarding the frequency of purchases, it should be admitted that respondents quite often buy organic food. As many as 38% of respondents buy at least once a month and 30% at least once a week (Figure 9).

**Figure 10:**
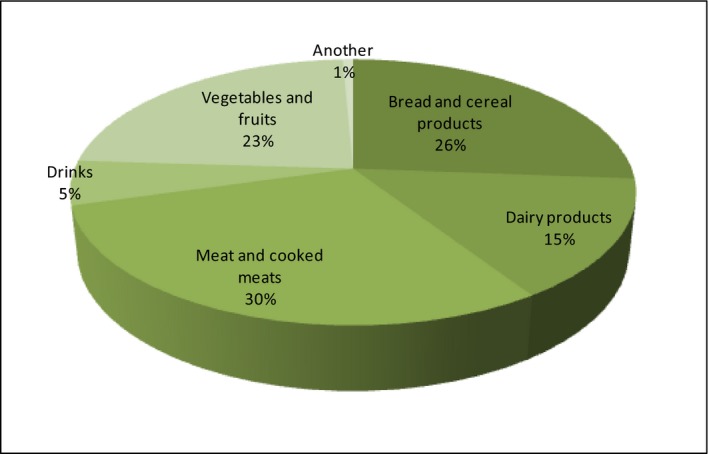
Types of organic food purchased by consumers

This is the most optimistic results proving that those who have declared the purchase of organic food do so quite regularly. Respondents have also expressed their preferences regarding the type of organic food they buy (Figure 10).

**Figure 11:**
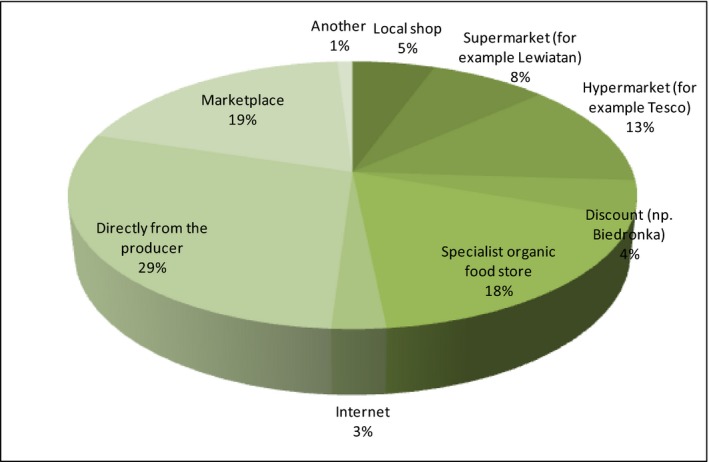
Places of purchase of organic food

The products such as meat and cooked meats (30%), bread and cereal products (26%), vegetables and fruits (23%), and dairy products (15%) have been mentioned in responses the most frequently. The Other category includes products such as sugar, oils, linseed oil, eggs, preserves, yoghurts, delicacies, dried fruits. There is also one more important issue related to shopping: the place of purchase. While the Internet has turned out to be one of the best sources of information about the product, unfortunately, it has not been appreciated as the place of purchase. Short food supply chains can be realized by different types of direct sale for instance, seasonal farmers’ market, consumer cooperatives and consumers (Sylla, Olszewska, Świąder, 2017). Next data refer to places of purchase of organic food (Figure 11).

It has turned out that the respondents prefer to buy organic food directly from the producer (29%), at the marketplace (19%), in a specialist organic food store (18%) and in a hypermarket (13%). Only 3% of respondents make purchases using the Internet.

The Other groups have included such statements as Krakowski Kredens, Rusiecki Kredens (brands offering food products), private owners, farms, herbal shop, and stalls in the shopping center. Therefore, it is clear from the presented data that among the preferred places for the purchase of organic food, the dominant way is the traditional one of getting rid of intermediaries (agricultural farm or marketplace).

### 4.3 Incentives for buying organic food

**Figure 12:**
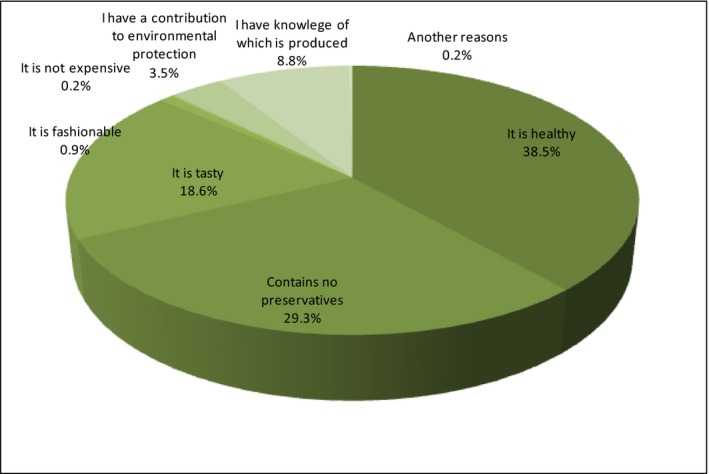
Reasons for the purchase of organic food

This part presents the motives of purchasing organic food among people who make such purchases and the reasons for not buying such food by the rest of the respondents. The reasons for the purchase of organic food to a predominant extent are attributed to health (38.5%), absence of preservatives (29.3%), taste (18.6%), and knowledge of which it has been produced (8.8%) (Figure 12).

**Figure 13:**
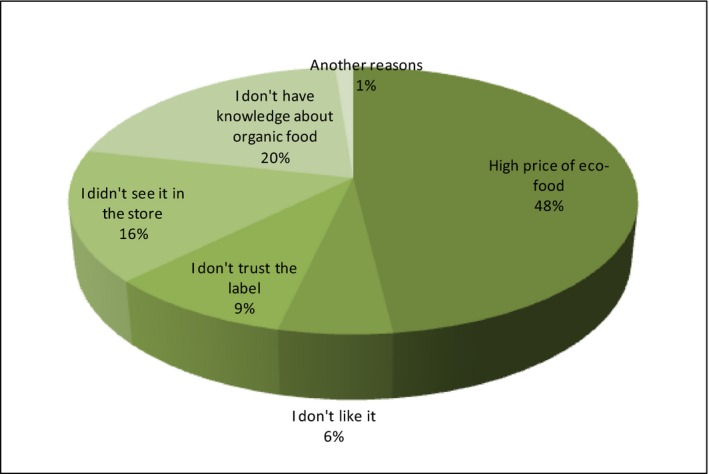
Reasons for not buying organic food

The remaining 46% of the respondents have been asked about the reasons for not buying organic food (Figure 13).

The vast majority of speakers consider a high price of eco‐food (48%) as the main decisive factor in abandoning the purchase. A lack of knowledge about this food (20%) has also been highly rated. As many as 16% of respondents admit that they do not meet organic food in the store. Among other reasons, they have mentioned: I did not think, I'm not interested, it does not matter to me, I do not know what organic food is, I am not convinced to such products, I think it is not worth its price.

The perception of prices, promotion and distribution on the bio‐food market

**Figure 14:**
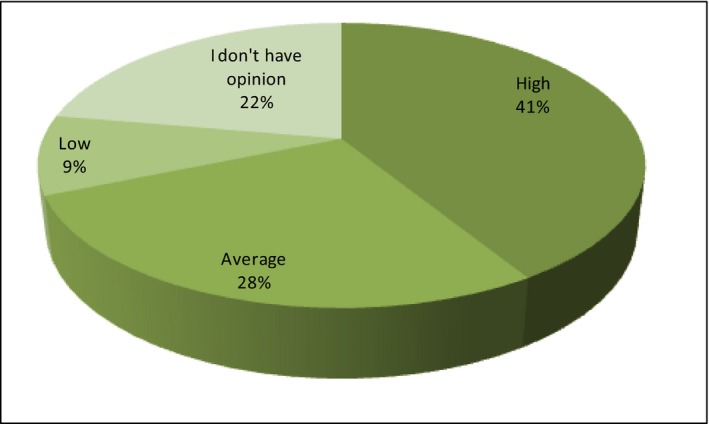
The level of prices of organic food in Poland in opinion of consumers

The price is the first analyzed element of perception of marketing activities. Therefore, the respondents have been asked how they assess the current price level of organic products. In most of the statements, the price level has been considered high (41%) or average (28%). Only 9% of respondents consider that the prices of organic products are low (Figure 14).

When the respondents treat high prices as a disincentive to buying, there is a presumption that with the reduction of these prices, the respondents would buy organic food. This is of course a hypothetical assumption. Therefore, the respondents have been posed before the following thesis: If the prices of organic food in Poland were equal to the prices of traditionally produced food, I would buy:

A more organic food than before
B the same amount of organic food than before
C less organic food than before
D I would buy only organic food
E I would not buy organic food at all
F I have no opinion

As many as 47.6% of respondents have stated that under the assumption of equal prices of organic and traditional food, they would buy more organic food than before. In an interesting manner, as many as 34.8% of respondents declare that they would buy only organic food then. Only 1.2% of the sample would not buy organic food at all, even after the price reduction. On the other hand, 6.3% of respondents would buy the same amount of food regardless of its price. About 9.7% of respondents are undecided. It can be clearly concluded that a change in the level of bio‐food prices would significantly change the demand for these products. People who have declared greater purchases of organic food are respondents with high (20.1%) and secondary education (20.5%), the place of residence in this group of people does not matter, because the distribution is almost even. A clear difference can be seen in the group of people who state that they would only buy organic food when prices are equal. They are the city residents up to 100,000 inhabitants (16.4%) with secondary education (18%). Those who want to buy more organic food than before (15.5%) and buyers of only organic food in the future (12.8%) belong to the group of respondents with incomes between PLN 1,501 and 2,500.

**Figure 15:**
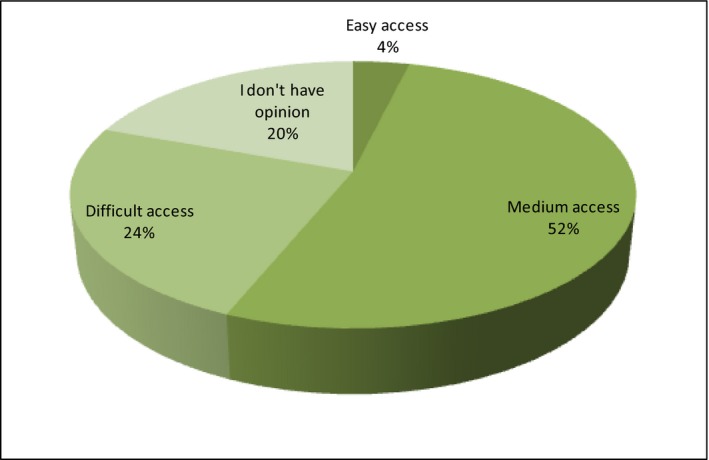
Availability of organic food in the stores

The last discussed area of research within the marketing of organic products is their distribution, and thus, availability on the Polish market. From the consumer's point of view, one should ask about availability in the store, as many of the respondents buy in the place of production of organic food (directly at the producer's). The producers themselves should be asked about the efficiency and effectiveness of distribution channels. The consumer is ultimately interested in the access to the product, so the respondents have been asked about the opinion on the access to organic products in the Polish stores. Most of the respondents rate availability as medium (52%) and even 24% as difficult (Figure 15).

So if this food is invisible to the consumer on the shelf, it should not be surprising that half of the respondents do not buy such food. About 20.4% of city dwellers up to 100,000 inhabitants consider organic food as moderately available. In an interesting manner, the inhabitants of the rural areas—19.2% and 9%—also consider organic food as medium and hard to reach. In the group of respondents earning PLN 1501–2500, there are consumers who consider organic food in Poland to be medium (16.3%) and hard (8.2%) available.

## 5 SUMMARY

The general objective of this work is to assess the development of the organic food market in Poland in terms of the selected marketing elements. To realize the main objective of the project, an empirical research has been conducted which results in formulating the following general conclusions:

- the range of organic food promotion in Poland is narrow and consumers expect price reductions as the most desirable form of promotion
- according to consumers, prices are too high and their reduction would influence on the increase in the number of purchased products
- in the opinion of consumers, organic food is hardly available in the Polish stores. Consumers prefer buying this food directly from the producer
- most consumers have heard about organic food and are looking for information about it in the Internet and on the product label. Over half of them have purchased such food during the last year
- as many as 44% of consumers admit to ignorance of the European logo of organic food and only 16% knowingly recognize the current labeling
- consumers correctly recognize the characteristics of the organic food production process and the properties of these products, which undoubtedly demonstrates awareness in this field
- consumers buy organic food on average once a month or once a week, and the most frequently they select products such as meat, sausages, bread, and cereal products as well as vegetables and fruits
- customers buy organic food because it is healthy and does not contain preservatives, taste values are not so important. A high price is the reason for refraining from buying it.
- the reason for the lack of interest in organic food by the consumer is ignorance about its labeling and high prices. However, consumers have satisfactory knowledge about the characteristics of organic food and the process of its production.

The survey conducted on a sample of consumers shows that almost all the people have heard about organic food. Half of the respondents have sought information about organic food.

Indicated problems and solutions require further and deeper analysis. Consumers are constantly learning and changing their attitudes. This may be challenging for researchers in this sector of the market. The obstacles of the development of the organic food market and the possible directions of development have been shown. The solution of these problems would bring many material benefits to the certified farmers and a better access to organic food for consumers. It is known, however, that the “eco‐consumer” on the Polish market is a well‐educated, middle‐income, and middle‐aged customer aware of the qualities of this food and the conditions accompanying the process of its production. These consumer features identified in the framework of the conducted research are information for the food producer who should get to know the interested customer. All data are available and the article was founded from private money.

## 6 STATEMENTS

1. A conflict of Interest statement, per the journal guidelines: https://onlinelibrary.wiley.com/page/journal/20487177/homepage/forauthors.html#_5._EDITORIAL_POLICIES. There is no conflict of Interest.
2. A statement that the study conforms to the Declaration of Helsinki, US, and/or European Medicines Agency Guidelines for human subjects—The study conforms.
3. A statement that the study's protocols and procedures were ethically reviewed and approved by a recognized ethical body (conform to Directive 2010/63/EU);—the study's protocols and procedures were ethically reviewed and approved by a recognized ethical body.
